# Prevalence and Awareness of Musculoskeletal Injuries Associated With Competitive Video Gaming in Saudi Arabia

**DOI:** 10.7759/cureus.37733

**Published:** 2023-04-17

**Authors:** Anas Fathuldeen, Mohammed F Alshammiri, Abdullah Abdulmohsen

**Affiliations:** 1 Medical Judicial Committee in Hail, Ministry of Health, Hail, SAU; 2 Plastic Surgery, College of Medicine, University of Hail, Hail, SAU; 3 College of Medicine, University of Hail, Hail, SAU

**Keywords:** saudi arabia, association, adults, musculoskeletal disorders, prevalence, video game

## Abstract

Background

In recent years, the use of electronic devices has become an important part of the lives of adolescents who regularly use computers for academic activities and leisure. The excessive use of these devices has been associated with several health problems, such as obesity, headaches, anxiety, stress, sleep disorders, and musculoskeletal pain. This study aimed to assess the prevalence and awareness of musculoskeletal injuries associated with competitive video gaming in Saudi Arabia.

Methodology

This descriptive, cross-sectional study targeted all individuals involved in competitive video gaming aged 18 years or older in Saudi Arabia. The data were collected using a researcher-initiated online questionnaire. The final electronic questionnaire contained questions on participants’ data, frequency and pattern of playing competitive video gaming, the associated musculoskeletal injuries, the most reported sites, and the associated consequences. The final questionnaire was sent to the participants via social media platforms and no more answers were obtained.

Results

A total of 116 competitive video gaming participants were included. Participants’ ages ranged from 18 to 48 years, with a mean age of 22.5. The majority of the participants were males (86.2%; 100). A total of 100 (86.2%) participants had at least one site-associated musculoskeletal injury, while only 16 (13.8%) had none. Regarding sites, the most reported sites included the lower back (63.8%), neck (50%), hand/wrist (44.8%), and shoulder (35.3%). A total of 58 (50.4%) thought that competing in electronic game tournaments negatively affects the musculoskeletal system, and 43 (37.1%) had an idea that competing in electronic gaming tournaments is linked to conditions such as tendinopathy, carpal tunnel syndrome, and repetitive stress injuries.

Conclusions

This study showed that the majority of competitive video gamers had musculoskeletal injuries mainly at the lower back, neck, hand/ wrist, and shoulder. A higher pain rate was reported among females and new gamers.

## Introduction

Video gaming is a widespread leisure activity, mainly among adults [1]. Video gaming attracts many people from different cultures to compete in organized video gaming events or tournaments at regional and international levels [2]. In these tournaments, professional and amateur players compete against one another in a competition form known as electronic sports (e-sports) [3]. The e-sports industry, including competitive video gaming (CVG), noticeably expanded with an estimated audience size of 532 million people worldwide and generated over 1.38 billion USD in revenue in 2022 [4].

Due to the rapid growth of players in the e-sports Industry, an increasing trend has been reported in the associated musculoskeletal injuries (MSIs) due to excessive training for competitions [5]. The most reported MSIs include neck and back, wrist, and hand pain [6]. Some studies have reported that hand and wrist pain is experienced among more than 60% of e-sport players [7,8]. Most upper limb injuries reported among e-sport players are mostly due to overuse injuries, including carpal tunnel syndrome [9] and tendinopathies in the shoulder and wrists [10]. Several upper limb injuries may affect a professional e-sports player. Any upper limb injury can be devastating to an e-sports player and may lead to significant career breaks, and, if ignored, may even be a career-ending injury. More than 400 fine motor movements per minute are performed by e-sports players with the shoulder, elbow, and wrist to stabilize the upper limb girdle using a mouse, keyboard, or handheld device [11]. Malposture associated with long hours of playing video games may increase the hazard of tendinopathies, nerve compression, and repetitive strain injuries [12]. This study aimed to contribute to e-sports-specific research by assessing the prevalence of MSIs related to a sedentary lifestyle, poor posture, and repetitive upper limb movements among competitive video gamers in Saudi Arabia. The study also sought to measure the awareness of competitive video gamers about these Injuries.

## Materials and methods

This descriptive, cross-sectional study targeted all individuals involved in CVG aged above 18 years in Saudi Arabia. According to Prince Faisal Bin Bandar Bin Sultan, chairman of the Sultan Saudi eSports Federation, the estimated number of people who play video games in Saudi Arabia is 23.5 million. As about 90% of them play video games as a hobby, this study targeted 10% who potentially engaged in CVG, which is equal to 2,350,000 million people. From this population, we calculated the sample size using an electronic sample size calculator, with an 8% margin of error, 90% confidence level, and 50% response distribution. The sample size was calculated at 106. The data were collected using a researcher-initiated online questionnaire after a comprehensive literature review and expert consultation. Competitive video gamers in Saudi Arabia who agreed to participate in the study were included. Participants with congenital skeletal disorders or musculoskeletal disorders secondary to chronic health problems were excluded. A panel of three experts reviewed the initiated questionnaire for validity and reliability, with all suggested modifications applied. The final electronic questionnaire contained questions on participants’ age, gender, education, employment, and body mass index (BMI). The second section covered participants’ frequency and pattern of playing competitive video games. The third section covered the associated MSIs, the most reported sites, and the associated consequences. The last section covered participants’ awareness regarding the effect of CVG-related disorders. The final questionnaire was sent to the participants via social media platforms until no more answers were obtained.

## Results

A total of 116 CVG participants were included. Participants’ ages ranged from 18 to 48 years, with a mean age of 22.5 years. The vast majority of the participants were males (86.2%; 100) and Saudi (66.4%; 77). Regarding educational level, 94 (81%) had a university-level education or above. A total of 71 (61.2%) of the participants were students, 25 (21.6%) were employed, and 20 (17.2%) were not employed. A total of 23 (19.8%) participants were overweight, and 21 (18.1%) were obese (Table 1).

**Table 1:** Personal data of competitive video gaming participants in Saudi Arabia.

Personal data	Number	%
Age in years		
18–20	51	44.0%
21–25	38	32.8%
26–30	20	17.2%
>30	7	6.0%
Gender		
Male	100	86.2%
Female	16	13.8%
Nationality		
Saudi	77	66.4%
Non-Saudi	39	33.6%
Educational level		
Secondary/below	22	19.0%
University/above	94	81.0%
Employment		
Unemployed	20	17.2%
Student	71	61.2%
Employed	25	21.6%
Body mass index		
Normal	72	62.1%
Overweight	23	19.8%
Obese	21	18.1%

Table 2 presents the CVG participation pattern among study respondents. A total of 78 (67.2%) of the respondents were participating in competitive video games for more than 10 years, 77 (66.4%) participated for about 20-30 days per month, 61 (52.6%) participated for four to six hours per day, and 28 (24.1%) participated for one to three hours per day. A total of 61 (52.6%) participants used PlayStation/Xbox for electronic gaming and 53 (45.7%) used PCs. Regarding practicing sports, 56 (48.3%) did but irregularly, while 28 (24.1%) did not practice at all.

**Table 2:** Competitive video gaming participation patterns among study respondents. CVG: competitive video gaming

CVG participation	Number	%
Duration of participating in competitive video games		
1–5 years	18	15.5%
6–10 years	20	17.2%
>10 years	78	67.2%
Participation days per month		
<10 days	12	10.3%
10–19 days	27	23.3%
20–30 days	77	66.4%
Participation hours per day		
1–3 hours	28	24.1%
4–6 hours	61	52.6%
7–9 hours	23	19.8%
10 or more hours	4	3.4%
What type of device do you use for electronic games?		
PlayStation/Xbox	61	52.6%
PC	53	45.7%
Mobile/tablet	2	1.7%
Do you practice sports?		
Five times/week	15	12.9%
Three times/week	17	14.7%
Irregularly	56	48.3%
Not practicing	28	24.1%

Table 3 presents the consequences of CVG among study participants. A total of 27 (23.3%) of the participants reported that they stopped participating in CVG due to injuries, only eight (6.9%) of them needed medical consultation due to injuries, six (5.2%) were diagnosed with a medical condition related to playing competitive video games, and 24 (20.7%) suffered from a bowed back (hump).

**Table 3:** Consequences of competitive video gaming among study participants. CVG: competitive video gaming

Consequences of CVG	Number	%
Stopped participating in electronic video gaming due to any injury		
Yes	27	23.3%
No	89	76.7%
Needed medical consultation due to any injury		
Yes	8	6.9%
No	108	93.1%
Have you ever been diagnosed with a medical condition related to playing video games?		
Yes	6	5.2%
No	110	94.8%
Do you suffer from a bowed back (hump)		
Yes	24	20.7%
No	92	79.3%

Table 4 summarizes the distribution of electronic gaming-associated MSIs by participants’ data. Only gender showed a significant association with associated MSIs. MSIs were reported among all female participants versus 84% of males, with recorded statistical significance (p = 0.048).

**Table 4:** Distribution of electronic gaming-associated MSIs by participants’ personal data. P: Pearson chi-square test; $: exact probability test; *: P < 0.05 (significant). MSI: musculoskeletal injury

Personal data	Musculoskeletal injuries				P-value
	Yes		No		
	Number	%	Number	%	
Age in years					0.548^$^
18–20	46	90.2%	5	9.8%	
21–25	32	84.2%	6	15.8%	
26–30	17	85.0%	3	15.0%	
>30	5	71.4%	2	28.6%	
Gender					0.048*
Male	84	84.0%	16	16.0%	
Female	16	100.0%	0	0.0%	
Educational level					0.162^$^
Secondary/below	21	95.5%	1	4.5%	
University/above	79	84.0%	15	16.0%	
Employment					0.545
Unemployed	17	85.0%	3	15.0%	
Student	63	88.7%	8	11.3%	
Employed	20	80.0%	5	20.0%	
Body mass index					0.330
Normal	64	88.9%	8	11.1%	
Overweight	20	87.0%	3	13.0%	
Obese	16	76.2%	5	23.8%	
Do you practice sports?					0.389^$^
Five times/week	12	80.0%	3	20.0%	
Three times/week	13	76.5%	4	23.5%	
Irregularly	51	91.1%	5	8.9%	
Not practicing	24	85.7%	4	14.3%	

Table 5 presents the distribution of electronic gaming-associated MSIs by respondents’ pattern of participation. All participants playing video games for one to five years had associated MSIs compared to 80.8% of those playing for more than 10 years (p = 0.045). Moreover, those playing for less than 10 days a month had MSIs in comparison to 83.1% of those playing for 20-30 days (p = 0.049). Others patterns were not significantly associated with having MSIs.

**Table 5:** Distribution of electronic gaming associated MSI by respondents’ pattern of participation P: Pearson chi-square test; $: exact probability test; *: P < 0.05 (significant). MSI: musculoskeletal injury

Participation pattern	Musculoskeletal injuries				P-value
	Yes		No		
	Number	%	Number	%	
Duration of participating in competitive electronic games					0.045*
1–5 years	18	100.0%	0	0.0%	
6–10 years	19	95.0%	1	5.0%	
>10 years	63	80.8%	15	19.2%	
Participation days per month					0.049*
<10 days	12	100.0%	0	0.0%	
10–19 days	24	88.9%	3	11.1%	
20–30 days	64	83.1%	13	16.9%	
Participation hours per day					0.786
1–3 hours	23	82.1%	5	17.9%	
4–6 hours	53	86.9%	8	13.1%	
7–9 hours	20	87.0%	3	13.0%	
10 or more hours	4	100.0%	0	0.0%	
What type of device do you use for electronic games?					0.352
Mobile/tablet	2	100.0%	0	0.0%	
PC	48	90.6%	5	9.4%	
PlayStation/Xbox	50	82.0%	11	18.0%	

**Figure 1:**
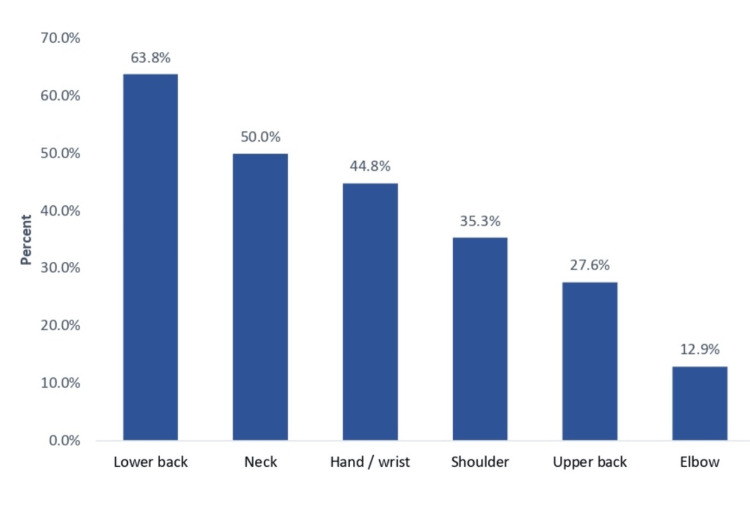
Sites of musculoskeletal injuries associated with competitive video gaming in Saudi Arabia.

Regarding the prevalence of MSIs associated with CVG in Saudi Arabia, 100 (86.2%) participants had at least one site-associated MSI, while only 16 (13.8%) had none. Regarding sites, the most reported sites included the lower back (63.8%), neck (50%), hand/wrist (44.8%), shoulder (35.3%), and upper back (27.6%), while the least reported site was the elbow (12.9%) (Figure 1).

**Figure 2:**
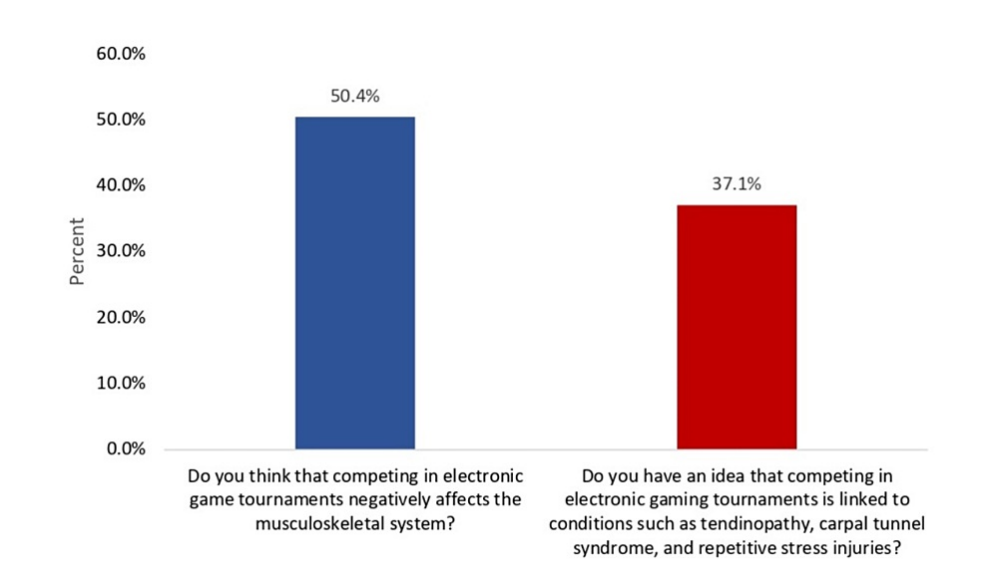
Awareness regarding musculoskeletal injuries associated with competitive video gaming in Saudi Arabia.

Figure 2 shows the awareness regarding MSIs associated with CVG in Saudi Arabia. Overall, 50.4% of the participants thought that competing in electronic game tournaments negatively affects the musculoskeletal system, and 43 (37.1%) had an idea that competing in electronic gaming tournaments is linked to conditions such as tendinopathy, carpal tunnel syndrome, and repetitive stress injuries.

## Discussion

A video game is defined as “a game which we play thanks to an audiovisual apparatus and which can be based on a story” [13]. Recently, studies focusing on video game playing have increased [14,15]. In addition to the popularity of gaming and e-sports in Saudi Arabia, e-sports tournaments and gaming competitions are also increasing. The Saudi Arabian Federation for Electronic and Intellectual Sports held its first e-sports and gaming tournaments in 2020 with more being planned. Other tournaments are hosted by private platforms such as the Saudi-based Kafu Games [16].

This study aimed to assess the prevalence and awareness of related MSIs among competitive video gamers in Saudi Arabia. The study revealed that most video gamers had at least one MSI. The most reported sites of MSIs among respondents were the lower back, neck, hand/wrist, and shoulder, whereas fewer injuries were reported at the upper back and elbow. Alnuman and Jbara [17] reported that video gamers reported moderate-to-severe discomfort in different body parts, mostly in the lower back (30%), upper back and neck (23%), and wrist (20%). Furthermore, Siu et al. [18] reported that more than two-thirds of computer users suffered from musculoskeletal discomfort. The shoulder (37.7%) and neck (35.0%) were the most commonly affected body parts for both genders, while female students reported higher rates of musculoskeletal discomfort, similar to the findings of this study. A systematic review by Tholl et al. [19] revealed a negative impact of video game playtime on the musculoskeletal system, with the most reported painful body parts being the neck (four studies), shoulder (four studies), and back (three studies). Silva et al. [20] concluded that more than two-thirds of adult video gamers experienced musculoskeletal pain symptoms which are more prevalent in the thoracolumbar spine (46.9%), followed by pain in the upper limbs (20%). Queiroz et al. [21] found that 61% of electronic device users had musculoskeletal pain. In Saudi Arabia, a study including gamers aged 18-25 years revealed that 6.7% of the respondents played computer games for more than 10 hours per day, and 11.1% played computer games 30 days per month. Regarding neck and back trauma, 10.3% reported previous neck injuries, whereas 27.2% reported previous back injuries [22]. Another study of Arabic children revealed that many children and adolescents experienced physical discomfort attributed to computer use ranging from neck pain (42%) to backache (30%) [23]. Another study on smartphone addiction and its relationship with MSIs showed that the prevalence of musculoskeletal disorders among addicts was 20.1% for the shoulder, 5.1 for the elbow, and 13.4% for the wrist/hand [24].

Regarding factors associated with having musculoskeletal disorders, this study showed that female gamers had higher a prevalence of musculoskeletal disorders than males. Other studies also reported similar findings [25,26]. Costigan et al. [27] found that girls are less physically active, with more time spent playing. Further, this study showed that the duration of playing video games and the days played per month were inversely related to musculoskeletal disorders, with new gamers showing a higher rate of having musculoskeletal disorders than those who played for more than 10 years. This may be explained by the fact that new gamers may experience high stress and more interaction, especially for competitive games than expert gamers, which may result in experiencing musculoskeletal discomfort. Many studies showed that play time may be associated with a higher incidence of MSIs, which is inconsistent with the findings of this study [28-30].

This study has some limitations. We did not include a control group in this cross-sectional study. We considered a margin of error of 8% at a 90% confidence level because we had limited access to the population due to economic and logistical reasons; therefore, the sample size was small. Only 13.8% of the participants were females, most likely reflecting that a minority of competitive video gamers are females; therefore, the results about females in our study might not be representative enough.

## Conclusions

This study showed that most competitive video gamers had MSIs mainly in the lower back, neck, hand/wrist, and shoulder. A higher pain rate was reported among females and new gamers who may experience more stress with the competition. Some video gamers stopped playing due to associated disorders, and one-fifth of them reported a bowed back (hump). Procedures to improve awareness and practice of the safe use of smartphones/online gaming, including postural education and decreasing screen time, are necessary.

## Appendices

1.   Sex*

o   Male

o   Female

2. Age (required over 18 years old)

……

3. Nationality 

o   Saudi

o   Non-Saudi

4. Educational level 

o   Primary

o   High school

o   College

o   Higher education (Master’s, PhD)

5. Employment status 

o   Employee

o   Unemployed

o   Student

6. Are you a participant or have you ever participated in a competitive electronic gaming tournament? (either formal or informal)

o   Yes

o   No

7. Weight in kilograms

……

8. Height  in centimeters:

……

9. Number of years of play

o   lLess than one year

o   1-5 years

o   6-10 years

o   More than 10 years

10. How many days do you play in a month?

o   Less than 10 days

o   10-19 days

o   20-30 days

11. How many hours do you play a day?

o   1-3 hours

o   4-6 hours

o   7-9 hours

o   More than 10 hours

12. What type of device do you use for electronic games?

o   PC

o   Playstation/Xbox

o   Mobile/tablet

13. Do you engage in physical activities?

o   Yes, 5 times a week

o   Yes, 3 times a week

o   Yes, infrequently

o   No

14. Do you think that competing in electronic gaming tournaments negatively affects the musculoskeletal system (joints, muscles, tendons, ligaments...)?

o   Yes

o   No

15. Do you have an idea that competing in electronic gaming tournaments is linked to conditions such as tendinopathy, carpal tunnel syndrome, and repetitive stress injuries?

o   Yes, I've heard of some/all of these cases

o   No, I've never heard of that before

16. Have you suffered from any problem (pain, roughness, numbness, tingling) in:*

the neck:

o   Yes

o   No

the shoulder:

o   Yes

o   No

elbow:

o   Yes

o   No

hand/wrist:

o   Yes

o   No

upper back:

o   Yes

o   No

lower back

o   Yes

o   No

17. Have you ever had an injury in the areas mentioned above that stopped you from playing for a while?

o   Yes

o   No

18. Have you ever gone to the doctor because of a problem (pain, roughness, numbness, tingling) in the areas mentioned above?

o   Yes

o   No

19. Have you ever been diagnosed with a medical condition related to playing electronic games?

o   Yes

o   No

20. If the answer is yes, what condition have you been diagnosed with?

…..

21. Do you suffer from a bowed back (hump)*

o   Yes

o   No
